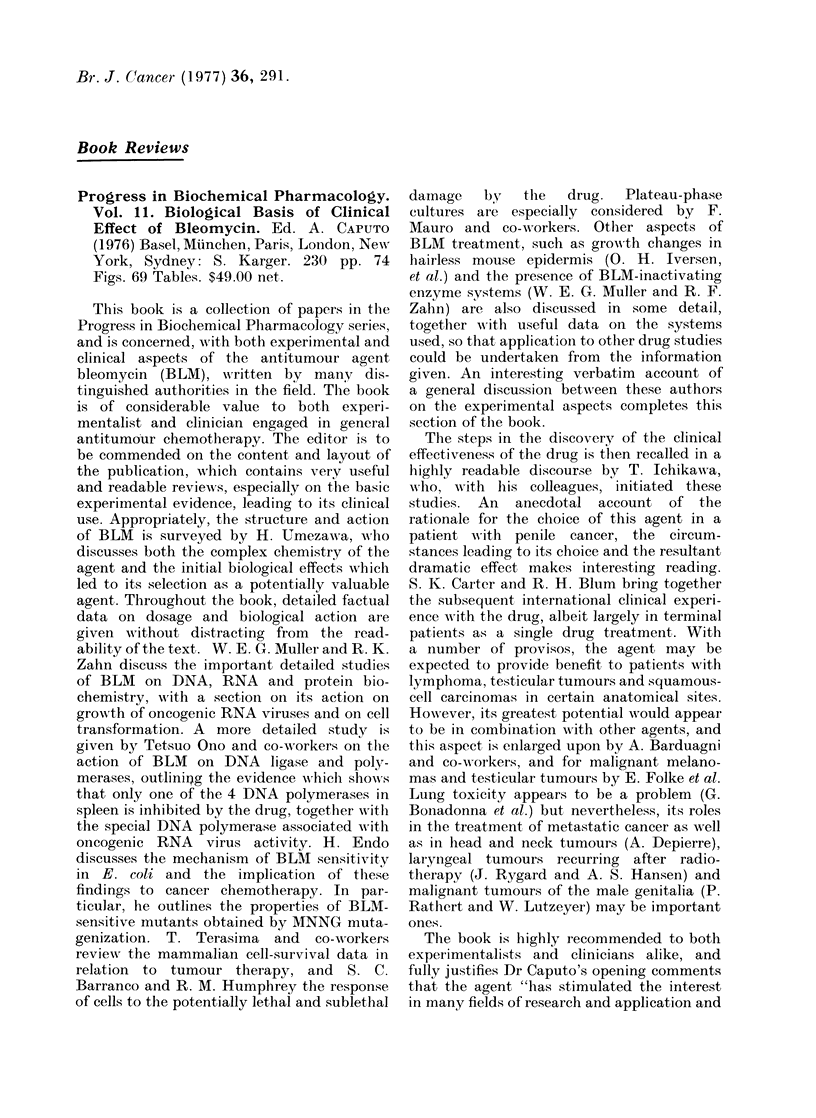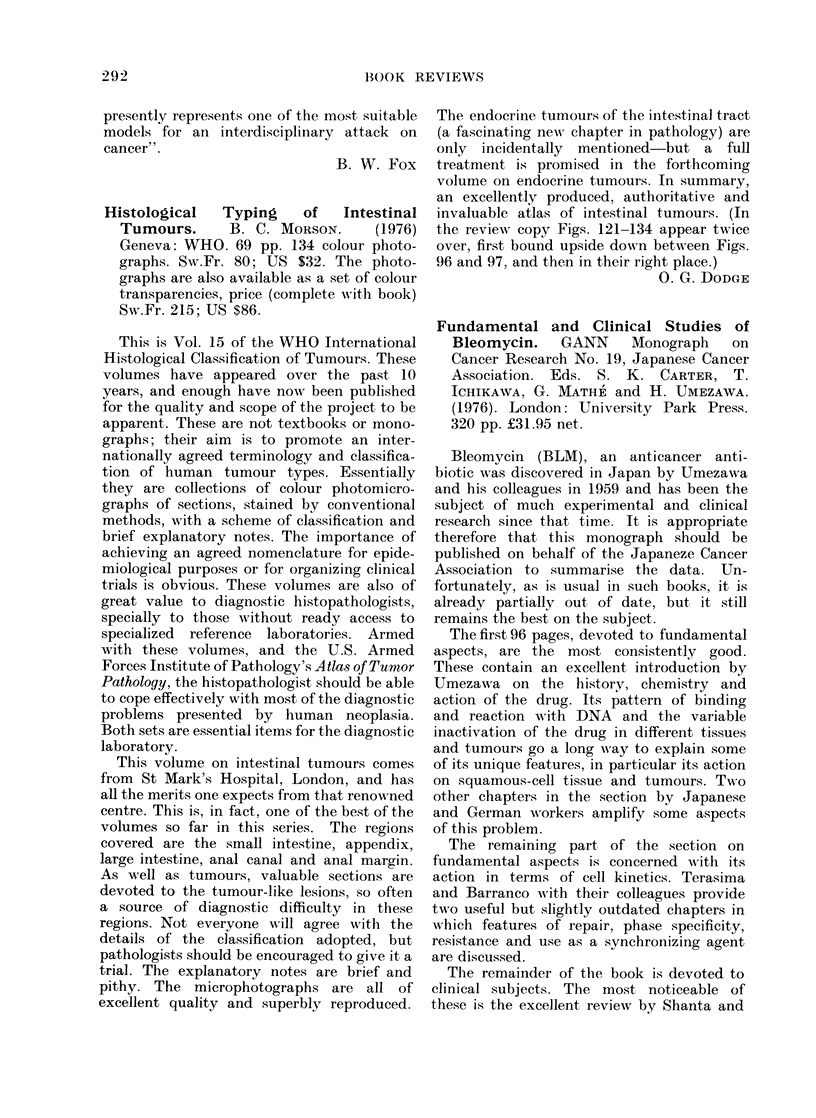# Progress in Biochemical Pharmacology Vol. 11. Biological Basis of Clinical Effect of Bleomycin

**Published:** 1977-08

**Authors:** B. W. Fox


*Br. J. Cancer* (1977) **36**, 291.

### *Book Reviews*

**Progress in Biochemical Pharmacology. Vol. 11. Biological Basis of Clinical Effect of Bleomycin.** Ed. A. CAPUTO (1976) Basel, München, Paris, London, New York, Sydney: S. Karger. 230 pp. 74 Figs. 69 Tables. $49.00 net.

This book is a collection of papers in the Progress in Biochemical Pharmacology series, and is concerned, with both experimental and clinical aspects of the antitumour agent bleomycin (BLM), written by many distinguished authorities in the field. The book is of considerable value to both experimentalist and clinician engaged in general antitumour chemotherapy. The editor is to be commended on the content and layout of the publication, which contains very useful and readable reviews, especially on the basic experimental evidence, leading to its clinical use. Appropriately, the structure and action of BLM is surveyed by H. Umezawa, who discusses both the complex chemistry of the agent and the initial biological effects which led to its selection as a potentially valuable agent. Throughout the book, detailed factual data on dosage and biological action are given without distracting from the readability of the text. W. E. G. Muller and R. K. Zahn discuss the important detailed studies of BLM on DNA, RNA and protein biochemistry, with a section on its action on growth of oncogenic RNA viruses and on cell transformation. A more detailed study is given by Tetsuo Ono and co-workers on the action of BLM on DNA ligase and polymerases, outlining the evidence which shows that only one of the 4 DNA polymerases in spleen is inhibited by the drug, together with the special DNA polymerase associated with oncogenic RNA virus activity. H. Endo discusses the mechanism of BLM sensitivity in *E. coli* and the implication of these findings to cancer chemotherapy. In particular, he outlines the properties of BLM-sensitive mutants obtained by MNNG mutagenization. T. Terasima and co-workers review the mammalian cell-survival data in relation to tumour therapy, and S. C. Barranco and R. M. Humphrey the response of cells to the potentially lethal and sublethal damage by the drug. Plateau-phase cultures are especially considered by F. Mauro and co-workers. Other aspects of BLM treatment, such as growth changes in hairless mouse epidermis (O. H. Iversen, *et al.*) and the presence of BLM-inactivating enzyme systems (W. E. G. Muller and R. F. Zahn) are also discussed in some detail, together with useful data on the systems used, so that application to other drug studies could be undertaken from the information given. An interesting verbatim account of a general discussion between these authors on the experimental aspects completes this section of the book.

The steps in the discovery of the clinical effectiveness of the drug is then recalled in a highly readable discourse by T. Ichikawa, who, with his colleagues, initiated these studies. An anecdotal account of the rationale for the choice of this agent in a patient with penile cancer, the circumstances leading to its choice and the resultant dramatic effect makes interesting reading. S. K. Carter and R. H. Blum bring together the subsequent international clinical experience with the drug, albeit largely in terminal patients as a single drug treatment. With a number of provisos, the agent may be expected to provide benefit to patients with lymphoma, testicular tumours and squamous-cell carcinomas in certain anatomical sites. However, its greatest potential would appear to be in combination with other agents, and this aspect is enlarged upon by A. Barduagni and co-workers, and for malignant melanomas and testicular tumours by E. Folke *et al.* Lung toxicity appears to be a problem (G. Bonadonna *et al.*) but nevertheless, its roles in the treatment of metastatic cancer as well as in head and neck tumours (A. Depierre), laryngeal tumours recurring after radiotherapy (J. Rygard and A. S. Hansen) and malignant tumours of the male genitalia (P. Rathert and W. Lutzeyer) may be important ones.

The book is highly recommended to both experimentalists and clinicians alike, and fully justifies Dr Caputo's opening comments that the agent "has stimulated the interest in many fields of research and application and presently represents one of the most suitable models for an interdisciplinary attack on cancer".

B. W. Fox